# Thirty Risk Factors for Alzheimer’s Disease Unified by a Common Neuroimmune–Neuroinflammation Mechanism

**DOI:** 10.3390/brainsci14010041

**Published:** 2023-12-31

**Authors:** Donald F. Weaver

**Affiliations:** Krembil Research Institute, University Health Network, Departments of Medicine, Chemistry, Pharmaceutical Sciences, University of Toronto, Toronto, ON M5T 0S8, Canada; donald.weaver@uhnresearch.ca

**Keywords:** Alzheimer’s disease, dementia, neurodegeneration, neuroinflammation, neuroimmune, microglia, cytokine

## Abstract

One of the major obstacles confronting the formulation of a mechanistic understanding for Alzheimer’s disease (AD) is its immense complexity—a complexity that traverses the full structural and phenomenological spectrum, including molecular, macromolecular, cellular, neurological and behavioural processes. This complexity is reflected by the equally complex diversity of risk factors associated with AD. However, more than merely mirroring disease complexity, risk factors also provide fundamental insights into the aetiology and pathogenesis of AD as a neurodegenerative disorder since they are central to disease initiation and subsequent propagation. Based on a systematic literature assessment, this review identified 30 risk factors for AD and then extended the analysis to further identify neuroinflammation as a unifying mechanism present in all 30 risk factors. Although other mechanisms (e.g., vasculopathy, proteopathy) were present in multiple risk factors, dysfunction of the neuroimmune–neuroinflammation axis was uniquely central to all 30 identified risk factors. Though the nature of the neuroinflammatory involvement varied, the activation of microglia and the release of pro-inflammatory cytokines were a common pathway shared by all risk factors. This observation provides further evidence for the importance of immunopathic mechanisms in the aetiopathogenesis of AD.

## 1. Introduction

The brain is the human body’s most complex and convoluted organ, and neurodegenerative disorders such as Alzheimer’s disease (AD) are arguably amongst the most complex diseases of the brain. One of the major hurdles encountered when formulating a mechanistic understanding with which to facilitate management strategies for AD is its immense complexity—a complexity that traverses the full structural and phenomenological spectrum, including molecular, macromolecular, cellular, behavioural and neurological processes [1,2]. AD risk factors are excellent examples of this immense complexity; these risk factors include such bewilderingly diverse conditions as medical diseases (diabetes), psychiatric disorders (depression), personal injuries (head trauma), societal factors (social isolation) and environmental issues (air pollution).

To identify a harmonizing mechanistic explanation with which to unify the many and varied risk factors for AD, a comprehensive literature review was initially completed (in PubMed, Web of Science, Scopus and Google Scholar databases including publications dating up to November 2023) and identified 30 “risk” factors for AD, employing a broad definition of “risk factor”: some are modifiable risk factors connected in a causative manner with AD (e.g., smoking, alcohol abuse, obesity); others are concomitant disorders occurring as co-morbidities (e.g., glaucoma; people with glaucoma are at risk for also developing AD); others are bidirectional risk factors (e.g., chronic pain causes neuroinflammation, which is a risk factor for AD, yet the neuroinflammation is a positive feedback risk factor for continuing pain). This comprehensive list of 30 risk factors includes the 12 modifiable risk factors identified in the 2020 Lancet commission (air pollution, alcohol abuse, brain injury, depression, diabetes, hearing impairment/deafness, hypertension, lower educational level, obesity, physical inactivity/sedentary lifestyle, smoking and social isolation) [3]. Beyond these 12 *Lancet* commission risk factors, 18 additional factors have been added, which include well-recognized risk factors that are non-modifiable (e.g., age, sex), risk factors that are modifiable but not at the personal level (e.g., climate change), concomitant co-morbidities as risks (e.g., glaucoma, migraine) and other newer factors for which convincing data are emerging but they remain less conclusive (e.g., oral hygiene, allergies).

Next, all literature sources discussing the 30 identified risk factors were searched for common terms providing mechanistic explanations. The term uniting all 30 risk factors was “neuroinflammation”, where neuroinflammation is defined as a functional process of the brain’s innate immune system following activation by diverse external (physical trauma, toxin (microbiological, chemical)) and/or internal (ischaemia) challenges, and manifesting as integrated cellular (microglial) and molecular (especially cytokine: e.g., Interleukin (IL)-1β, IL-6 and Tumour Necrosis Factor (TNF)-α) alterations within the brain [4,5]. Since many studies provide data strongly implicating neuroinflammation as a significant contributor and culprit in the aetiopathogenesis of AD, a shared neuroimmune–neuroinflammation mechanism clearly emerges as a unifying thread providing harmonization within the rich tapestry of diverse risk factors associated with AD.

**Figure 1:**
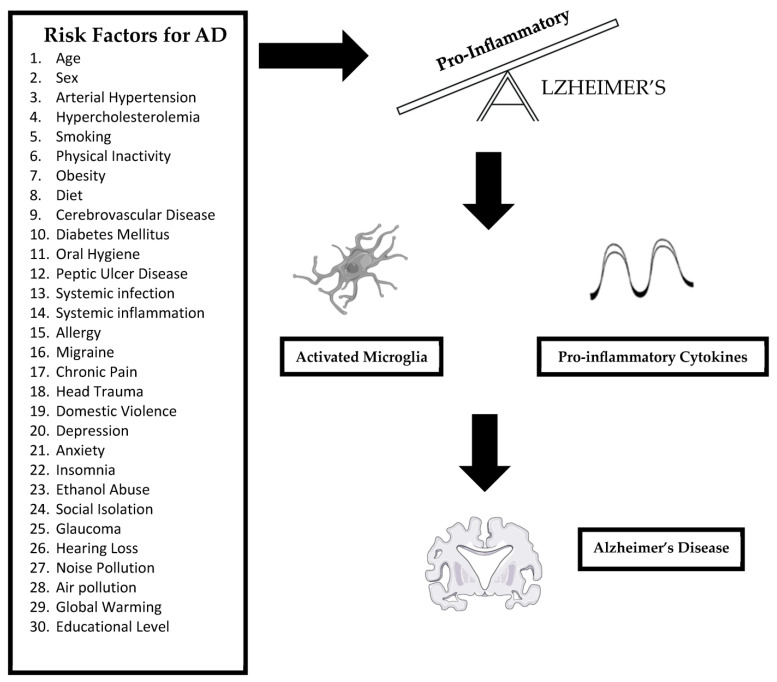
Thirty risk factors for Alzheimer’s disease: traditionally AD was regarded as a proteopathic disease arising from protein misfolding and aggregation; however, immunopathy also contributes to AD particularly as an excessive pro-inflammatory innate immune response. The 30 very diverse risk factors for AD identified in this review are uniquely unified by their common ability to elicit neuroinflammation, manifesting as microglial activation and pro-inflammatory cytokine (IL-1β, IL-6, TNFα) release, ultimately causing neuronal loss and brain atrophy thereby contributing to the pathogenesis of the disease. These risk factors cause an imbalance in immune homeostasis triggering excessive pro-inflammatory activities which are neurotoxic.

Herein, an overview of the neuroimmune–neuroinflammation axis as related to AD is presented followed by a consideration of the 30 risk factors for AD in conjunction with a description of their neuroinflammatory mechanisms (Figure 1).

### Neuroimmune–Neuroinflammatory Contributions to Alzheimer’s Disease

Traditionally, AD has been regarded as a proteopathy (i.e., protein-based disorder) arising from the misfolding and oligomerization of β-amyloid (Aβ) and tau. Regrettably, however, this conceptualization has failed to yield a definitive curative therapy, thereby necessitating the need to explore other mechanistic approaches, including immunopathy, gliopathy, mitochondriopathy, membranopathy, synaptotoxicity, metal dyshomeostasis and oxidative damage, reflecting the biochemical complexity and heterogeneity of AD. Of these mechanisms, immunopathy is emerging as a lead contender [6,7,8,9,10].

Not surprisingly, an immunopathic mechanistic explanation of AD is likewise complex and involves a host of cellular (microglia) and molecular (cytokine) participants. Emerging data indicate that the homeostatic balance between pro-inflammatory and anti-inflammatory processes becomes disordered over the time duration of the disease, ultimately tilting towards a neuropathic pro-inflammatory milieu and manifesting with increased concentrations of activated microglia and pro-inflammatory cytokines (IL-1β, IL-6, TNFα) [6,7,8]. During the initial pre-symptomatic phases of the disease, immune processes are neuroprotective with microglia-mediated phagocytosis of cytotoxic Aβ aggregates. However, as the disease progresses, such neuroprotective effects are supplanted by neurotoxic effects with elevated pro-inflammatory processes. These neurotoxic pro-inflammatory effects occur within the context of both innate immunity and adaptive immunity, with deleterious neuroinflammation arising primarily from the actions of prolonged innate immunity activity. Neuroinflammation involves reactive, pro-inflammatory microglia and astrocytic phenotypes, which paradoxically enhance Aβ oligomerization and promote tau hyperphosphorylation, complement activation and the catabolism of neurotransmitters and brain biomolecules into neurotoxic metabolites—changes which both initiate and/or propagate neurodegeneration, heralding cognitive reduction and dementia in susceptible (usually geriatric) adults. Since the neurotoxicity of excessive pro-inflammatory processes occurs not only at the level of innate immunity, via neuroinflammation, but also at the level of adaptive immunity, the neuropathological mechanisms of neuronal death involve both auto-inflammatory and autoimmune mechanisms. Additional support for the aetiopathogenic role of the neuroimmune–neuroinflammation axis AD comes from genetic studies: genome-wide association studies (GWASs) reveal that multiple polymorphisms associated with AD occur in genes that regulate innate immune function (e.g., CD33, CLU, CR1, TREM-2), which encode proteins that regulate complement activation and cellular phagocytic activities [9]. Thus, although inflammation, in general, is a non-specific response to many different types of injury, within the specific context of AD, the neuroimmune–neuroinflammation axis is a key contributor to disease pathogenesis and progression; accordingly, factors that affect the biochemistry or histology of this axis emerge as risk factors for AD [10].

## 2. Thirty Risk Factors

### 2.1. Age

Although AD is not a normal long-term outcome of aging, age is regarded as the best established risk factor for the disease. The number of people living with AD doubles every five years after age 65 years; 40% of people aged 90 years and older have AD [11]. In the preponderance of people diagnosed with AD, symptoms onset after they reach their mid-60 s in age or even later. When the disease manifests clinically before age 65, it is regarded as uncommon.

The links between aging and AD are many and complex; however, neuroinflammation is a key component of this link, with aging being associated with neuroinflammation and neuroinflammation being associated with AD. D’Avila et al. established that aged mice exhibit dystrophic pro-inflammatory microglia in the entorhinal cortex and hippocampus within the medial temporal lobe [12]. Aged mice also release higher levels of pro-inflammatory (IL-1β and IL-6) cytokines in the brain and higher levels of NADPH oxidase 2 (Nox2) expression compared to younger animals [13].

In humans, aging and a chronic inflammatory state frequently co-exist in the periphery and in the brain. Aging impairs functional interactions between the brain and the immune system; microglia and astrocytes, functioning in their capacity as innate immune cells, become more pro-inflammatory during aging [14]. This age-associated increase in innate immune reactivity heralds an augmented inflammatory cytokine brain response after activation of the innate immune system during the initiation and progression of AD, leading to more severe long-lasting behavioural and cognitive deficits.

### 2.2. Sex

After age, sex is the other most commonly cited risk factor for AD. Women are more likely to develop dementia over the course of their life (even after greater longevity is considered); twice as many women have AD compared to men. A Swedish study by Beam et al. followed 16,926 people and noted that commencing at age 80 years, women are more likely to be identified as having AD than men at corresponding ages [15]. An analogous Taiwanese study by Liu et al. concluded that the likelihood of developing AD throughout a seven-year time duration was greater in women compared to men [16]. Finally, a meta-analysis by Niu et al. studying the European incidence of AD calculated that, annually, 13 women out of 1000 developed AD, compared to only 7 men [17].

Immune-mediated neuroinflammatory responses are different between men and women. Women are more susceptible to inflammatory pathological consequences than men via neuroimmune alterations, including microglial activation, pro-inflammatory cytokine expression and dysinformational synaptic plasticity [18]. In a study involving injecting volunteers with immunogenic lipopolysaccharides (LPSs), Engler et al. ascertained that women undergo a significantly enhanced pro-inflammatory response, with higher circulating levels of TNFα and IL-6; conversely, the LPS-triggered rise in anti-inflammatory IL-10 was significantly greater in men [19]. Finally, women constitute >80% of all diagnoses of autoimmunity, particularly as demonstrated by differences in the incidence for Sjögren syndrome, systemic lupus erythematosus, Hashimoto thyroiditis, Graves’ disease, scleroderma and myasthenia gravis [20]; Meier-Stephenson et al. argued that AD is an autoimmune disease. Such sex-based neuroimmune differences provide a possible explanation for the corresponding sex differences in the incidence and prevalence of AD [21].

### 2.3. Arterial Hypertension

Hypertension is a well-documented and accepted risk factor for AD. Multiple studies have concluded the existence of a correlation between cognitive decline and systemic arterial hypertension in different age cohorts [22,23]. Systemic arterial hypertension, particularly midlife high blood pressure (BP), has been related to a higher risk of dementia, including AD. In the middle years of life (age 40–64 years), there is a positively correlated relationship between BP elevation and cognitive dysfunction in AD, whilst in elderly populations (age ≥ 65 years), this relationship is more controversial, with hypotension being deleterious to intellectual function.

Not surprisingly, the link between hypertension and AD is multifactorial, with vascular factors playing a major contributing role. However, neuroinflammation is another major mechanism linking hypertension and AD [24]. Animal studies have established that prolonged BP elevation culminates in neurotoxic glial activation and increased cerebral inflammatory mediators, particularly pro-inflammatory cytokines, such as TNFα and IL-1β. Solé-Guardia et al. observed that individuals experiencing chronic hypertension had an enhanced neuroinflammatory response, manifesting as augmented microglial activation and astrogliosis and more apparent perivascular inflammation compared to non-hypertensives [25]. Carnevale et al. showed that hypertension induced microglial activation, and interleukin IL-1β upregulation triggers neuroinflammation before Aβ deposition [26].

### 2.4. Hypercholesterolemia

Dysregulated cholesterol biosynthesis and metabolism constitute a risk factor for AD and multiple other diseases. In vivo and human-based investigations have concluded that a high-cholesterol diet (HCD) induces A. In rats and mice, HCD produces significant cognitive decline and AD-like disease [27,28]; in Japanese white rabbits on an HCD, alterations in brain structure and function analogous to those of human AD were noted [29]. Epidemiological investigations have also suggested a relationship between hypercholesterolemia and AD [30]. Xu et al. suggested that high cholesterol levels were associated with increased AD pathology severity, and that the mechanism for this enhanced pathology is not entirely mediated by cerebrovascular conditions [31]. Thus, mounting evidence indicates that excessive cholesterol accumulates in AD, driving AD-associated pathological changes, and that hypercholesterolemia promotes AD development as a risk factor, especially with elevated cholesterol levels in the middle years of life.

As with hypertension, the link between hypercholesterolemia and AD is multifactorial, with vascular factors playing a major contributing role. However, neuroinflammation is another major mechanism linking hypercholesterolemia and AD [32]. For example, Thirumangalakudi et al. demonstrated that hyperlipidemic mice showed increased expression of pro-inflammatory microglia and cytokines/mediators, including TNFα, IL-1β, IL-6, NOS2 (Nitric oxide synthase 2) and COX2 (Cyclooxygenase-2) [33]. Chen et al. also showed, in mice, that a high-cholesterol diet enhanced pro-inflammatory NLRP3 (NLR family pyrin domain containing 3) inflammasome activation and IL-1β expression [34].

### 2.5. Smoking

Based on a comprehensive review, Durazzo et al. concluded that smoking tobacco products gives rise to a significantly intensified risk for AD and dementia [35]. Cigarette smoke/smoking is associated with AD neuropathology in both preclinical models and human studies. Jeong et al. showed that smoking discontinuation resulted in a reduced risk of dementia [36].

The negative consequences of smoking are numerous, providing multiple mechanisms by which smoking contributes to pathology. However, immune-based inflammation is a significant contributor to this pathology. Alrouji et al. concluded that smoking inflicts complex immunological effects, which include enhancements in inflammatory responses (activated microglia with increased pro-inflammatory cytokine responses) with a concomitant lessening of immune defences, causing an increased vulnerability to the deleterious effects of a chronic ongoing pro-inflammatory environment [37]. In a case–control study, Liu et al. found that cigarette smoking was associated with elevated concentrations of at-risk biomarkers for AD, as indicated by higher neuroinflammation biomarkers in the cerebrospinal fluid of participants in the active smoking group [38].

### 2.6. Physical Inactivity

Based on a comprehensive literature review, Meng et al. concluded that physical inactivity is one of the most readily addressable and avoidable risk factors for AD and that improved physical activity levels are linked to a diminished risk of AD development [39]. Physical exercise is also helpful in improving behavioural and psychiatric symptomatic indicators of AD, notably via better cognitive function. Chen et al., likewise, concluded that physical exercise is important in the prevention of AD, providing non-pharmacological treatment options [40].

The case correlating physical inactivity with AD via a neuroinflammatory mechanism is strong. Recently, Wang et al. demonstrated that exercise ameliorates AD by directly and indirectly regulating brain immune responses and promoting hippocampal neurogenesis [41]. Similarly, Seo et al. showed that neuroinflammation-mediated microglia activation with pro-inflammatory cytokine release is enhanced by physical inactivity and downregulated by exercise [42]. Svensson and co-workers likewise showed that exercise leads to the elevated biosynthesis and release of anti-inflammatory cytokines, and lower concentrations of pro-inflammatory cytokines and activated microglia [43].

### 2.7. Obesity

Obese people exhibit a higher risk of acquiring age-correlated cognitive reduction, mild cognitive impairment and AD [44,45]. An association between body mass index (BMI) and AD has been described, with multiple groups studying the relationship between elevated BMI and AD. Obesity is, thus, a recognized risk factor for AD [46,47,48,49].

Miller and Spencer suggested that neuroinflammation is the linkage that unites obesity with AD; obesity (and high fat consumption) culminates in systemic inflammation as well as elevated levels of circulating free fatty acids and inflammatory mediators. These circulating cytokines and activated immune cells reach the brain and initiate local neuroinflammation, including microglial proliferation and causing synaptic remodelling and neurodegeneration [50]. Similarly, Henn et al. also suggested that immune dysregulation, including inflammaging (e.g., age-related increase in the levels of cytotoxic pro-inflammatory biomarkers in blood and tissues) and immunosenescence (e.g., age-related reduction in the efficacy of immune system function), commonly occur prematurely as a consequence of obesity, promoting cognitive impairment and AD [51].

### 2.8. Dietary Factors

In recent years, numerous studies have confirmed that, especially with advancing age, diet affects cognitive capacities and ultimate susceptibility to AD. In a systematic search of randomized clinical trials, reviews and meta-analyses evaluating the connection between diet and AD, Xu Lou et al. examined 38 studies and concluded that a Western diet pattern is a risk factor for AD, whereas the Mediterranean diet, ketogenic diet and supplementation with omega-3 fatty acids and probiotics are potentially neuroprotective diets [52]. The Mediterranean diet, the related MIND diet (which incorporates constituents designed to lower blood pressure) and other healthful dietary regimens are associated with cognitive benefits in studies and a decreased probability of AD [53,54,55,56].

Kip and Parr-Brownlie noted that many dietary risks factors are linked to AD-promoting neuroinflammation, particularly high saturated and trans-fat intake; indeed, dietary modifications in mice can influence levels of pro-inflammatory microglia and cytokines [57]. Conversely, dietary restriction (DR) has been shown to diminish age-related pro-inflammatory activation of microglia, astrocytes and cytokines while prolonging lifespan in various organisms [58,59].

The microbiome also plays an essential role in the link between diet and AD. Dietary changes (either deleterious or beneficial) influence the microbiome composition, thereby altering the gut–brain homeostatic axis with the release of pro-inflammatory bacterial metabolites, which predispose people to AD progression [60].

### 2.9. Cerebrovascular Disease

Cerebrovascular disease, manifesting as cerebral atherosclerosis and arteriolosclerosis, is a risk factor associated with AD; thus, cerebral vasculopathy is a pervasive risk factor for both vascular dementia and AD [61]. A number of midlife vascular risk factors are significantly associated with AD—findings consistent with a role of vascular disease in the development of AD [62]. Stroke is a common pathology associated with AD among elderly individuals—a co-morbid relationship at its fullest when accompanied by a plethora of commonly acknowledged vascular risk factors [63]. Vascular risk factors associated with AD include the conventionally recognized risk factors (hypertension, cholesterolemia) which contribute to atherosclerotic vascular change, as well as amyloid angiopathy, in which amyloid deposits in the walls of small to medium cerebral blood vessels lead to microhaemorrhages with consequent neurologic deficits, which may include impairments in memory or cognition.

Beyond the obvious vascular contributions (ischaemia, hypoxemia) to dementia, neurotoxic brain inflammation (pro-inflammatory microglia and cytokines) accompanies the ischaemic conditions of cerebrovascular disease, thereby contributing to AD pathogenesis [64,65,66]. Jurcau and Simion showed that neuroinflammatory mechanisms significantly contribute to neuronal injury during cerebral ischemia, ultimately further increasing the extent of cerebral damage and neurological deficits in AD [67].

### 2.10. Diabetes Mellitus

Numerous studies have shown that people with diabetes, especially Type 2 Diabetes, are at higher risk for AD; indeed, AD has even been referred to as Type 3 Diabetes [68,69,70]. Among people with diabetes, the risk for AD is 65% higher than in non-diabetic controls. Conversely, but analogously, in people with AD, the prevalence of diabetes is higher than anticipated, approaching 35%. An even greater number of people with AD (46%) have glucose intolerance, which is often a metabolic predictor of diabetes. Even without overt clinically demonstrated diabetes, dysregulation of the glucose metabolism is associated with cognitive decline and AD risk.

Given the complexity of diabetes, the possible mechanistic links between diabetes and AD are multi-fold and include Aβ misfolding and oligomerization, tau hyperphosphorylation and aggregation, neuroinflammation, damaging pro-oxidative processes and dysfunctional mitochondria. Amongst these, Van Dyken and Lacoste argued that neuroinflammation is one of the key mechanistic connectors between diabetes and AD [71]. Similarly, based on a systematic review of in vitro, preclinical and clinical studies, Vargas-Soria et al. concluded that diabetes triggers specific responses that include the upregulation of activated microglia and secretion of a wide variety of pro-inflammatory cytokines and chemokines [72]. Pathways commonly activated by diabetic pathological changes include the NLRP3 inflammasome.

### 2.11. Oral Hygiene (Porphyromonas gingivalis)

Bacteria and their associated inflammatory molecules are able to travel from regions of mouth infections to the brain via the bloodstream [73]. Researchers in the School of Dentistry, University of Central Lancashire, initially drew attention to the link between oropharyngeal disease and AD, concluding that periodontitis/gingivitis is a risk factor for AD [74]. The mouth contains 700 bacterial species, including ones that cause periodontal gingivitis; *Porphyromonas gingivalis*, a Gram-negative, rod-shaped, pathogenic anaerobic bacterium from the phylum Bacteroidota, is the most common culprit of gum disease. Recent studies indicate that Aβ oligomerization and its associated neuroinflammatory responses may be triggered in response to this infection. *Porphyromonas gingivalis* and the gingipains enzyme which it produces have been identified in AD brains. Thus, periodontitis is an anatomically specific infection and risk factor for AD [75,76].

**Figure 2:**
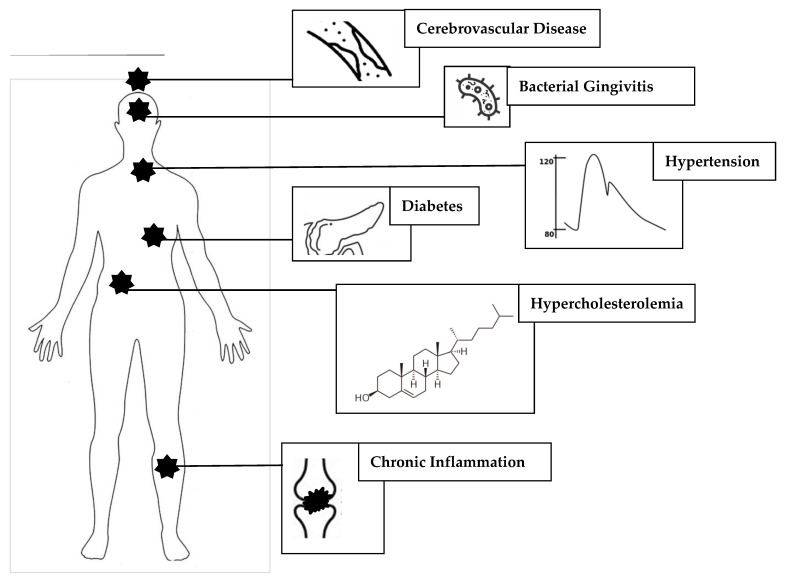
Risk factors for Alzheimer’s disease: although AD is a disease of the central nervous system, the diverse risk factors that contribute to its initiation and progression are not confined to the brain and often are systemic disorders such as diabetes mellitus, arterial hypertension or chronic inflammation. Non-systemic localized factors, anatomically distinct from brain, such as chronic periodontitis/gingivitis dental inflammation, are also emerging risks. May of the risk factors are interconnected (e.g., vascular disease, hypertension, hypercholesterolemia and diabetes) and mutually self-sustaining; these factors also contribute to the pathogenesis of AD via a multi-factorial route, through not only neuroinflammatory processes, but also vascular dysfunction.

Neuroinflammatory processes constitute the connection between chronic, inflammatory disease of the oropharyngeal cavity and gums (periodontitis) and AD [77]. This neuroinflammation may occur through two basic processes: a. local (oral) and/or its associated systemic inflammation, triggering a neuroinflammatory reaction within the brain via the distribution of pro-inflammatory mediators; or b. direct entry of bacteria into the cranial space, eliciting a protective innate immune response manifesting as neuroinflammation. Also, pathogenic oropharyngeal bacteria release structurally diverse metabolites and inflammatory mediators into the bloodstream, ultimately crossing the brain–blood barrier (BBB); these bacteria can instigate alterations in gut microbiota, further enhancing inflammation and affecting brain function via the gut–brain axis. The fifth cranial (trigeminal) nerve has been proposed as an alternative route for connecting oral bacterial products to the brain. Whatever the mechanism, periodontitis/gingivitis leads to microglial activation and pro-inflammatory cytokine release in the brain, thereby triggering and promoting AD pathogenesis [78,79] (see Figure 2).

### 2.12. Peptic Ulcer Disease (Helicobacter pylori)

There is also an association between peptic ulcer disease and AD, analogous to the connection between oral bacteria and AD, but with the peptic ulcer bacterium (Helicobacter pylori) being further down the gastrointestinal tract [80,81]. Studies have shown that peptic ulcer disease increases the risk of AD via the mechanisms of systemic inflammation and altered gut microbiota [82]. In a population-based study, Chang et al. showed that the suppression of Helicobacter pylori yields decreased progression of dementia [83]. Thus, periodontitis and peptic ulcer disease are two anatomically specific infections implicated as risk factors for AD.

Noori et al. showed that Helicobacter pylori infection contributes to the expression of AD-associated risk factors and neuroinflammation, particularly enhanced concentrations of activated microglia and pro-inflammatory cytokines [84]. In a rat model of peptic ulcer disease, increased levels of circulating pro-inflammatory cytokines such as IL-1β were documented [85].

### 2.13. Systemic Infection

The relationship between systemic non-CNS infections and AD is complex, but a preponderance of evidence supports the supposition that systemic infection is a risk factor for AD [86]. Giridharan et al. showed that infection-induced systemic sepsis accelerates cognitive decline and neuropathology in an AD mouse model [87]. Based on a systematic review and meta-analysis of human studies, Lei et al. showed that surviving sepsis was linked to a greater risk of dementia (OR = 1.62, 95% CI = 1.23–2.15, I^2^ = 96.4%, *p* = 0.001) and that septicaemia is associated with increased risk for dementia and AD [88]. Though many microorganisms have been implicated, Herpes simplex virus 1, Chlamydia pneumonia and Borrelia burgdorferi have been discussed as infectious agents, which are possible specific microbiological risk factors for AD. Conversely, systemic infection exacerbates pre-existing AD, accelerating cognitive decline and disease progression.

Systemic infections provoke a systemic inflammatory response, which, in turn, elicits neuroinflammation. In a prospective human pilot study, Holmes et al. demonstrated that cognitive function is negatively impacted for two months or longer following the resolution of a systemic infection and that elevated serum levels of IL-1β herald this cognitive impairment [89]. In a post-mortem study, Asby et al. provided evidence that systemic infection raises the levels of multiple cytokines (TNFα, IL-1β, IL-6, IL-8 and IL-15) in the brain [90].

### 2.14. Systemic Inflammation

Acute and chronic systemic inflammation is characterized by the systemic production of pro-inflammatory cytokines (e.g., TNFα) that play a role in immune to brain communication; systemic inflammation increases pro-inflammatory cytokine secretion in the brain, which, in turn, causes an increase in cognitive decline and disease progression in AD [91]. Walker et al. discussed how systemic pro-inflammatory cytokines can traverse the BBB and enter the brain to regionally promote a pro-inflammatory environment, via a process which also involves signalling through endothelial cells and/or activating the vagus nerve [92]. Systemic inflammation, thereby, induces phenotypically reactive pro-inflammatory microglia and astrocytes, which further can foster β-amyloid oligomerization, tau hyperphosphorylation and complement activation. Similarly, Xie et al., likewise, discussed how peripheral inflammation is a risk factor contributing to AD by means of neuroinflammation [93]. Finally, diseases typically associated with chronic systemic inflammation, such as rheumatoid arthritis, are regarded as risk factors for AD [94,95].

### 2.15. Allergies

Joh et al. studied 6,785,948 adults aged ≥40 years who participated in a national health examination without any history of dementia before baseline; during 8.1 years of follow-up, 260,705 dementia cases (195,739 AD) were identified, and three allergic diseases (asthma, atopic dermatitis, allergic rhinitis) were positively associated with dementia risk [96]. Compared with individuals without allergies, those with all three allergic diseases had a substantially increased risk of AD (multivariable hazard ratios 1.46; 95% CI 1.25–1.70). Bożek et al. also noted a similar correlation between allergies and AD [97]. Conversely, allergies can exacerbate existing health issues for older adults with AD.

Not surprisingly, there is a relationship between allergies and inflammation [98]. Kabata and Artis described how allergies affect a variety of cytokines, inflammatory mediators and neuropeptides to yield an enhanced neuroinflammatory response [99]. Similarly, Mirotti et al. extensively reviewed the relationship between allergies and brain inflammation, particularly microglial activation and pro-inflammatory cytokine release [100].

### 2.16. Migraine Headache

In a nationwide (South Korea) cohort study, Kim et al. showed that the overall incidence of AD was greater in people with a chronic migraine history than in non-migraineurs (8.0 per 1000 person-years vs. 4.1 per 1000 person-years) [101]. Similarly, in a population-based cohort study involving 88,390 participants, Hurh et al. concluded that migraine is associated with an increased risk of subsequent AD [102]. Multiple other epidemiological studies support the observation that migraine is a risk factor for AD [103,104].

Migraine is a neuroinflammatory disorder [105], with evidence of neuroinflammation in vascular and perivascular spaces. The implications of co-existing migrainous neurogenic inflammation and neuroinflammation in the histochemical pathophysiology of migraine have been repeatedly demonstrated in preclinical models involving dural vessels and trigeminal endings within the trigemino-vascular system. Neuroinflammatory pathways, especially those invoking inflammasome protein involvement, are regarded as clinical biomarkers and promising druggable targets for migraine [106].

### 2.17. Chronic Pain

In a France-wide propensity-matched cohort group, Bornier et al. noted that among 64,496 people, the incidence of AD was higher in the chronic pain population than in a control group (1.13% vs. 0.95%, *p* < 0.001); chronic pain increases the risk of AD [107]. Supportively, in a systematic review, Innes and Sambamoorthi documented the possible involvement of chronic pain to cognitive impairment and subsequent dementia including AD [108]. Also, Cao et al. provided evidence that supports a risk factor link between chronic pain and AD [109].

In mechanistic considerations, Vergne-Salle and Bertin discussed how sensory peripheral nerve fibres conveying pain messages are able to mediate peripheral sensitization processes, which, in turn, are linked to the elaboration of inflammation molecules; these afferent nerve fibres trigger neurotransmitter release in spinal cord dorsal root ganglia and dorsal horns, thereby activating microglia and producing pro-inflammatory cytokines and chemokines throughout the CNS [110]. Moreover, as with many of these risk factors, the relationship is bidirectional, self-sustaining and mutually triggering, as evidenced by the fact that neuroinflammation enhances chronic pain perception [111].

### 2.18. Head Trauma

Young adults who experience moderate to severe head trauma have a greater-than-two-fold enhanced risk for developing AD or a related dementia later in life [112]. In a study based on a population-based prospective historical cohort design, Plassman et al. showed that both moderate head injury (hazard ratio (HR) = 2.32; CI = 1.04 to 5.17) and severe head injury (HR = 4.51; CI = 1.77 to 11.47) were associated with an increased risk of AD [113]. Thus, there is evidence for an association between a history of previous head injury and the risk of developing AD.

Schimmel et al. showed that neuroinflammation following traumatic brain injury is a chronic response to an acute injury [114]. Simon et al. demonstrated that some individuals with traumatic brain injury develop chronic neuroinflammation, which can last for years after the injury, and is associated with activated microglia and the release of pro-inflammatory cytokines—a conclusion also supported by Xiong et al. and Zheng et al. [115,116,117].

### 2.19. Domestic Violence

Intimate partner violence (IPV; also termed spousal abuse or domestic violence) forms a sub-group of head trauma scenarios uniquely correlated with AD [118]. However, IPV is more than a focussed sub-type of head trauma. Unlike the head trauma typically seen during accidents or in professional athletes, IPV also comprehensively encompasses psychological, sexual and financial abuse and, not infrequently, is accompanied by alcohol or substance abuse; the nature of the physical violence in IPV is also different, frequently involving manual or ligature partial strangulation.

A 1990 case report by Roberts et al., describing a 76-year-old woman with dementia, connected IPV and AD [119]. A woman was found unconscious with head contusions; her relatives disclosed that her husband had been abusive for years. A post-mortem brain examination revealed morphological and immunological characteristics showing that the woman’s IPV-associated brain trauma contributed significantly to the development and progression of her dementia. The consequences of traumatic brain injuries (TBIs) are significant, with evidence suggesting a single TBI may double one’s likelihood of developing dementia. Traumatic brain injuries are highly prevalent amongst victims of IPV, arguably leaving hundreds of millions of women worldwide at increased risk for developing dementia.

The connection between IPV and AD is clear and involves multiple mechanisms including neuroinflammation. Newton et al. showed that IPV histories are associated with biologic mediators of inflammation, particularly elevated levels of IL-6 [120]. Similarly, Madison et al. showed that IPV is associated with augmented pro-inflammatory cytokine responses including IL-6 and TNFα [121].

### 2.20. Depression

Arguably, depression and dementia (AD) share a continuum as a single spectrum disorder: depression leads to dementia and dementia leads to depression. Depression is, thus, a risk factor for AD—an assertion supported by multiple studies. Moreover, emerging evidence is indicating that the time-point in life during which the depression occurs is crucial in determining the nature of this mutually triggering association between AD and depression. In particular, earlier-life depression is associated with a more-than-doubled increase in risk for AD and related dementias; in contrast, analyses of geriatric-onset depression are less definitive but, in general, they too support the notion of a depression–dementia co-dependency [122]. A variety of studies support these conclusions that depression is a risk factor for AD [123,124,125,126,127,128].

Multiple studies suggest that neuroinflammation is the key process linking depression to dementia [129]. In depression, chronic activation of innate immunity accelerates central inflammation, leading to higher levels of inflammatory cytokines, most consistently IL-1β, IL-6 and TNFα, which, in turn, correlates with greater depressive symptomatology [130]. Neuroinflammation is involved in the pathophysiology of depression through the actions of pro-inflammatory cytokines, which influence interneuronal cross-talk via serotonergic pathways as well as neurogenesis and neuroplasticity in mood-related cerebral regions; these cytokines also stimulate the hypothalamus–pituitary–adrenal axis, exerting influence on hormonal-mediated mood alterations [131].

### 2.21. Anxiety

Based on a comprehensive literature review, Becker et al. concluded that anxiety is a risk factor for AD (*n* = 26193, hazard ratio 1.53, 95% CI 1.16–2.01, *p* < 0.01) [132]. Similarly, based on a meta-analysis of prospective cohort studies, Santabárbara et al. evaluated nine prospective cohorts representing 29,608 participants and identified an overall relative risk of dementia of 1.24 (95% CI: 1.06–1.46) and a population fraction of dementia attributable to anxiety of 3.9%; they concluded that anxiety is extensively connected with an enhanced risk for AD [133].

The relationship between anxiety, neuroinflammation and AD is complex and bi-directional: anxiety causes neuroinflammation and neuroinflammation causes anxiety (analogous to the depression–dementia spectrum). Studies by Won and Kim suggest that anxiety disrupts the hypothalamic–pituitary–adrenal axis and affiliated autonomic nervous system activities; in turn, this mutually induces enhanced pro-inflammatory cytokine levels from activated microglia, particularly in prefrontal and limbic brain structures. The resulting enhanced neuroinflammatory conditions contribute to AD progression [134]. Conversely, based on animal and clinical studies, Zheng et al. and Guo et al. concluded that neuroinflammation induces anxiety by modulating neuronal plasticity in multiple brain regions but, particularly, the basolateral amygdala [135,136]. Thus, anxiety triggers neuroinflammation central to the pathogenesis of AD.

### 2.22. Insomnia

Sleep disorders, including insomnia, are a well-documented risk factor for AD [137]. In general, neurodegenerative diseases cause sleep disruption, also exemplified by clinical events such as “sundowning” and nocturnal wandering; conversely, chronic insomnia is itself a risk factor for neurodegenerative diseases, including AD. This is not surprising given that sleep has important roles in learning and memory consolidation. Also, sleep deprivation affects not only the symptoms but also the molecular pathogenesis of AD. Sleep contributes to the sequestration and removal of Aβ from neural tissue: Kang et al. showed in transgenic mice that chronic insomnia leads to Aβ accumulation and symptomatic disease progression [138]. Thus, multiple studies have now convincingly demonstrated that sleep deprivation is a risk factor for AD [139,140,141,142,143,144].

Neuroinflammation is the central cellular and molecular connection between insomnia and AD. Zhu et al. showed that disturbed sleep architecture increased pro-inflammatory IL-6 cytokine levels and induced the phenotypic activation of microglia in the mouse hippocampus, impairing learning and memory, which are hippocampus-dependent processes [145]. Zielinski and Gibbons described the neurotoxic pro-inflammatory role of the IL-1β and TNFα inflammatory cytokines and the NLRP3 inflammasome during periods of dysregulated sleep [146]. Chronic insomnia has also been associated with compromised structural integrity of the BBB, which permits increased entry of peripheral immune cells (macrophages) and inflammatory cytokines into the CNS, further contributing to the ongoing neuroinflammation implicated in AD pathogenesis [147]. Therefore, sleep impairment leads to neuroinflammation through increasing levels of pro-inflammatory cytokines (TNFα, IL-6 and IL-1β) and enzymes (COX), which catalyse inflammatory neurochemical processes.

### 2.23. Ethanol Abuse

Alcoholism is a substance abuse disorder clinically associated with multiple and varied cognitive problems, including acute intoxication, delirium, Wernicke’s psychosis, alcoholic dementia and AD. Not surprisingly, chronic ethanol abuse has been identified as a risk factor for cognitive decline, AD and dementia [148]. Ethanol is a small lipophilic molecule capable of altering multiple neurochemical pathways, which subserve the cognition and memory processes essential to normal brain function; chronic ethanol toxicity, thus, shares and enhances negative effects on normal brain psychology with AD. In turn, this justifies the assertion that alcohol abuse increases the risk of developing AD [149,150,151,152,153].

Neuroinflammation is a major histochemical component of alcohol-induced neural damage [154]. Alcohol abuse triggers peripheral inflammation and central neuroinflammation; the receptor-mediated enabler of this diffuse inflammatory response is the upregulation of the innate immunity TLR4 (Toll-Like Receptor 4) protein with subsequent microglial and inflammatory cytokine involvement. Based on mouse studies, Lowe et al. established that chronic ethanol use and abuse promote the pathological entry of peripheral macrophages into the brain, with accompanying microglia activation mediated by stimulation of the CCR2/5 (C-C chemokine receptor types 2 and 5) immune receptor axis [155].

### 2.24. Social Isolation

Loneliness and social isolation are widespread and significant public health risks affecting many people and placing them at enhanced risk for AD. In an analysis of 502,506 British Biobank participants and 30,097 Canadian Longitudinal Study of Aging participants, Shafighi et al. evaluated risk factors for developing AD in the context of loneliness and aberrant social networking support; they identified strong links between social isolation and AD [156]. Similarly, in a study to establish Cox proportional hazard models with social isolation and loneliness as separate exposures, Shen et al. concluded that social isolation is a risk factor for AD that is independent of loneliness [157].

Neuroinflammation is a definite immunological concomitant of the psychosocial problems inflicted by social isolation. In a study on eight-week-old male C57BL/6 mice, Al Omran et al. showed that social isolation resulted in microglial activation and the release of pro-inflammatory cytokines [158]. Analogously, in a study with BALB/c mice, Ayilara and Owoyele demonstrated evidence of neuroinflammation manifesting as increased activated microglial count and elevated IL-1β and TNFα cytokine levels in a social isolation rearing model [159]. Also, Vu et al. showed that social isolation produces brain region-specific activation of the microglia state in C57Bl/6 mice [160].

### 2.25. Glaucoma

Glaucoma is the collective diagnostic term for a group of ocular diseases characterized by optic neuropathies linked to degeneration of the retinal ganglion cells; though glaucoma is conventionally conceptualized as a disorder of intraocular pressure, it is better regarded as primarily a disorder of neural tissue within the optic nerve, leading to visual impairment and blindness. Evidence of a link between AD and glaucoma has arisen from epidemiological analyses, revealing that people with AD have a significantly increased incidence of glaucoma [161]. Cesareo et al. studied 51 AD subjects and 67 sex-matched controls: subjects underwent measurements of intraocular pressure, visual field testing and retinal nerve fibre layer thickness assessment by slit-lamp biomicroscopy—patients with AD had a higher frequency of glaucoma-like alterations [162]. Crump et al. studied 324,730 persons diagnosed with glaucoma from 1995 to 2017 in Sweden and 3,247,300 age- and sex-matched population-based controls without prior dementia: in 16 million person-years of follow-up, 32,339 (10%) persons with glaucoma and 226,896 (7%) controls were diagnosed with dementia [163]. Persons with glaucoma had increased risks for AD (adjusted HR, 1.39; 95% CI, 1.35–1.43); among glaucoma subtypes, both primary open-angle and normal-tension glaucoma was associated with an increased risk for AD. Thus, people with glaucoma have an increased risk of developing AD [164,165].

Preclinical and clinical evidence supports the notion that glaucoma is a widespread neurodegenerative condition, whose shared pathogenic mechanism with AD is neuroinflammation. Williams et al. showed that the neuropathology of glaucoma extends beyond the visual pathways and involves pro-inflammatory neuroinflammation at both a cellular (microglia, astrocyte) and molecular (cytokine) level in other CNS locations [166]. Studies by Rolle et al., Rutigliani et al. and Soto and Howell reached similar conclusions [167,168,169].

### 2.26. Hearing Loss

Hearing loss at ages 45–65 is a significant risk factor for dementia, possibly accounting for 8 percent of all dementia cases; a 2020 *Lancet* report determined that hearing loss across a wide variety of types and aetiologies approximately doubles the risk of dementia, with even subclinical hearing loss enhancing AD risk [3]. Extensive studies by Lin et al. concluded that hearing loss is associated with increased cognitive decline and incident AD and other dementias in older adults [170]. Based on an analysis of a UK biobank cohort, Jiang et al. concluded that in people with hearing loss, restorative hearing aid use is associated with a reduced risk of dementia of a similar level to that of people without hearing loss, thereby highlighting the urgent need to take measures to address hearing loss as a remediable risk factor for AD [171].

Seicol et al. showed that age-related hearing loss is accompanied by chronic inflammation in neural structures, with elevated expression of pro-inflammatory cytokines and microglial activation [172]. Similarly, Frye et al. demonstrated that pro-inflammatory cytokines including TNFα and IL-1β, and chemokines including CCL2, are induced by hearing loss [173].

### 2.27. Noise Pollution

Despite their obvious interconnection, hearing loss and exposure to noise pollution are regarded as separate risk factors. Hearing loss caused by factors other than noise exposure is a risk factor for AD; chronic noise exposure of insufficient magnitude to cause obvious hearing loss is, likewise, a risk factor for AD. Epidemiological studies are increasingly identifying the association between external noise exposure (via noise pollution) and dementia [174]. Weuve et al., for example, showed that an increment of 10 A-weighted decibels (dBA) in noise corresponded to 36% and 29% higher odds of prevalent mild cognitive impairment (MCI; odds ratio (OR) = 1.36; 95% confidence interval (CI), 1.15 to 1.62) and AD (OR = 1.29, 95% CI, 1.08 to 1.55) [175]. Cantuaria et al. estimated that as many as 1216 out of the 8475 cases of dementia registered in Denmark in 2017 could be attributed to noise exposures, indicating a great potential for dementia prevention through reductions in ambient noise such as that arising from roadway traffic [176].

As with hearing loss, neuroinflammation is a central mechanistic player in the pathogenesis of noise-induced AD. Wang et al. showed that noise exposure is associated with elevated expression of pro-inflammatory cytokines and microglial activation in the primary auditory cortex; genetic knockout of TNFα or pharmacologically blocking TNFα expression prevented this neuroinflammation [177]. Similarly, Cui et al. showed that chronic noise exposure acts cumulatively to exacerbate neuroinflammation and AD pathology in the rat hippocampus [178].

### 2.28. Air Pollution

Based on a systematic literature review, Peters et al. concluded that greater exposure to PM2.5, NO2/NOx and CO was associated with an increased risk of dementia, where PM2.5 is airborne particulate matter ≤2.5 μ in size [179]. Subsequently, Peters and Li reaffirmed this observation, claiming that constituents of PM2.5, namely black carbon, organic matter, sulphates (SO_4_^2−^) and ammonium (NH_4_^+^), from traffic and fossil fuel combustion are significantly associated with the development of AD [180]. Also, a national cohort study (2000–2018) of long-term air pollution exposure and incident dementia in older adults in the United States showed that exposures to PM_2.5_ and NO_2_ are associated with an increased incidence of AD [181,182].

Campbell et al. showed that exposure to particulate matter in polluted air increases biomarkers of inflammation in the mouse brain, including activated microglia, and levels of pro-inflammatory cytokines such as IL-1β and TNFα [183]. Tin-Tin-Win-Shwe et al., likewise, showed changes in pro-inflammatory cytokine mRNA expressions in mice following nanoparticle air pollution exposure [184]. These data and others led Block and Calderón-Garcidueñas to conclude that the emerging evidence implicates air pollution as a chronic source of neuroinflammation, instigating AD with activation of microglia as key to this process [185].

### 2.29. Global Warming

In 2021, the World Health Organization (WHO) announced that climate change is the biggest global health threat to humanity’s future. A 1.5 °C ambient temperature increase may seem trivial when one considers diurnal and seasonal variations, but it does induce subtle but tangible effects on neural pathways and mechanisms that underlie normal brain functioning; these pathways, including neuroinflammation, are implicated in neurodegeneration [186]. Thus, it is possible that global warming secondary to climate change will emerge as a risk factor for AD by facilitating a state of chronic neuroinflammation. In addition, climate warming puts people with AD at risk for symptom worsening and disease progression [187,188,189]. Gong et al. predicted a 4.5% increase in the risk of dementia hospital admission per 1 °C increase above 17 °C and a 300% increase in hospital admissions for AD by 2040 because of climate change [190]. Although risk factors such as diet and obesity are personally modifiable, risk factors such as climate change are problems which require societal solutions at an international level (Table 1).

As with the other risk factors, neuroinflammation is a key consideration in ascribing a mechanistic explanation for climate change as an AD risk factor. Given the relationship between ambient temperatures and inflammation, it is probable that neuroinflammation is part of the pathological spectrum response to global warming [191,192]. For example, in mice subjected to heat exposure, Lee et al. found: (1) an increased number of glial fibrillary acid protein (GFAP)- and macrophage-1 antigen (Mac-1)-positive cells, (2) up-regulated nuclear factor (NF)-κB, a master regulator of inflammation, and (3) marked increases in COX-2, inducible nitric oxide synthase (iNOS),and cytokine IL-1β and TNFα in the mouse hippocampus [193].

### 2.30. Educational Level

Lower education is associated with a greater risk for AD and related dementias [194]. The 2020 *Lancet* Commission that examined dementia risk factors found 7% of worldwide dementia cases could be prevented by increasing early-life education [3]. This analysis also concluded that higher childhood education levels and higher lifelong educational attainment could reduce AD and dementia risk. A focussed sub-type of educational attainment is the ability to speak multiple languages; multiple studies indicate that bilingualism or multilingualism offer a degree of protective delay against the development of AD [195,196,197].

The correlation of educational level with neuroinflammation is not as immediately apparent as for other risk factors, such as head trauma. Nonetheless, there are data clearly supporting a relationship between education and brain inflammatory markers. Steinvil et al. found a statistically significant inverse association between number of school years and high-sensitivity C-reactive protein (CRP), fibrinogen and erythrocyte sedimentation rate (ESR), concluding that level of education was inversely associated with inflammatory biomarkers, even within highly educated populations [198]. Similarly, Maurel et al. found a relationship between educational attainment and five inflammatory biomarkers (CRP, fibrinogen, IL-1β, IL-6 and TNFα), whereby a low educational attainment was associated with higher inflammation, even after adjusting for health behaviours and body mass index [199]. A 2015 study by Okonkwo and co-workers showed that older adults who completed at least 16 years of education had less evidence of AD biomarkers in their cerebrospinal fluid (CSF) than people with fewer years of education [200].

However, education is a complex societal phenomenon. Thus, it is also possible that education is associated with a higher socioeconomic status and quality of life (i.e., less obesity, better diet, better access to healthcare for hypertension, diabetes, depression, deafness) that helps keep people healthy and lowers AD risk.

## 3. Conclusions

The development of effective diagnostics and therapeutics for AD is one of humankind’s pressing neuropharmacologic priorities. A hurdle in the successful attainment of these priorities is the immense cellular and molecular complexity of AD. This complexity is reflected by the equally complex diversity of risk factors associated with AD. However, more than merely mirroring disease complexity, risk factors also provide fundamental insights into the aetiology and pathogenesis of AD as a neurodegenerative disorder since they are central to disease initiation and subsequent propagation. Based on a systematic literature review, this analysis identified 30 risk factors for AD and then extended the analysis to further identify neuroinflammation as a unifying mechanism present in all 30 risk factors. Although other mechanisms (e.g., vasculopathy) were present in multiple risk factors, dysfunction of the neuroimmune–neuroinflammation axis was key to all 30 identified risk factors. Though the nature of the neuroinflammatory involvement varied, activation of microglia and the release of pro-inflammatory cytokines were common pathways shared by all risk factors. This observation provides further evidence for the importance of immunopathic mechanisms to aetiopathogenesis of AD.

Neuroinflammation is “bad for brain”. The identification of these 30 risk factors for neuroinflammation (and, therefore, AD) is, thus, also a call to action. By 2050, more than 150 million people will be living with AD—the health and socioeconomic impacts of this statistic will be truly immense. Since humanity is struggling to devise curative therapeutics for AD, prophylactically addressing risk factors is and will continue to be an essential step in reducing the global burden of AD. This review identified 30 risk factors. Some are modifiable and can be addressed at the level of the individual (depression, diabetes, diet, educational level, excessive alcohol consumption, hearing impairment, hypertension, low social contact, obesity, oral hygiene, peptic ulcer disease, physical inactivity, smoking, traumatic brain injury); others need to be addressed at a societal or international level (air pollution, climate change, noise pollution, intimate partner violence). Meaningfully addressing these risk factors requires multi-level educational goals, targeting individuals, healthcare providers, school teachers, politicians and policy makers. Hopefully, we—individually and collectively—have the commitment to attain these goals, thereby reducing the neuroinflammation that mediates the transformation of lifestyle/societal circumstances into risk factors for a devastating disease.

## Figures and Tables

**Table 1:** Risk factors for AD.

Constitutive Factors
Age (neuroinflammation, proteopathy, vascular)
2. Sex (neuroinflammation, proteopathy, vascular)
Personal Modifiable Factors
3. Arterial Hypertension (vascular, neuroinflammation, proteopathy)
4. Hypercholesterolemia (vascular, neuroinflammation, proteopathy)
5. Smoking (vascular, neuroinflammation, proteopathy)
6. Physical Inactivity (vascular, neuroinflammation, proteopathy)
7. Obesity (vascular, neuroinflammation, proteopathy)
8. Diet (vascular, neuroinflammation, proteopathy)
9. Diabetes Mellitus (vascular, neuroinflammation, proteopathy)
10. Cerebrovascular Disease (vascular, neuroinflammation, proteopathy)
11. Oral Hygiene (neuroinflammation, proteopathy)
12. Peptic Ulcer Disease (neuroinflammation, proteopathy)
13. Head Trauma (trauma, neuroinflammation, proteopathy)
14. Depression (neuroinflammation, proteopathy)
15. Anxiety (neuroinflammation, proteopathy)
16. Insomnia (neuroinflammation, proteopathy)
17. Ethanol Abuse (neuroinflammation, proteopathy)
18. Social Isolation (neuroinflammation)
19. Hearing Loss (neuroinflammation)
Societal Modifiable Factors
20. Domestic Violence (trauma, neuroinflammation, proteopathy)
21. Noise Pollution (neuroinflammation)
22. Air pollution (neuroinflammation)
23. Global Warming (neuroinflammation)
24. Educational Level (neuroinflammation, proteopathy)
Comorbidity or Concomitant Risk Factors
25. Systemic infection (neuroinflammation)
26. Systemic inflammation (neuroinflammation)
27. Chronic Pain (neuroinflammation)
28. Chronic Migraine (neuroinflammation, proteopathy)
29. Chronic Allergies (neuroinflammation)
30. Glaucoma (neuroinflammation, proteopathy)

## Data Availability

Not applicable.
